# Niemann-Pick type C: contemporary diagnosis and treatment of a classical disorder

**DOI:** 10.1136/practneurol-2019-002236

**Published:** 2019-06-26

**Authors:** Meher Lad, Rhys H Thomas, Kirstie Anderson, Timothy D Griffiths

**Affiliations:** 1 Department of Clinical Neurosciences, Royal Victoria Infirmary, Newcastle upon Tyne, UK; 2 Institute of Neuroscience, Newcastle University Medical School, Newcastle upon Tyne, UK

**Keywords:** metabolic disease, neurogenetics, eye movements, cerebellar ataxia, dementia

## Abstract

Niemann-Pick type C is an uncommon neurodegenerative lysosomal storage disorder that can cause a progressive neuropsychiatric syndrome associated with supranuclear vertical gaze palsy and a movement disorder. There have been recent developments in testing that make diagnosis easier and new therapies that aim to stabilise the disease process. A new biochemical test to measure serum cholesterol metabolites supersedes the skin biopsy and is practical and robust. It is treatable with miglustat, a drug that inhibits glycosphingolipid synthesis. We describe a patient, aged 22 years, with juvenile-onset Niemann-Pick type C who presented with seizures and a label of ‘cerebral palsy’. We describe the approach to this syndrome in general, and highlight the classical features and red flags that should alert a neurologist to this treatable condition.

## Case presentation

A 29-year-old woman was referred to us with a progressive syndrome. There was no neurological disease in her family; however, there had been no contact with her biological father since his mid-thirties. She had been born at term with no perinatal insult, hypoglycaemia or jaundice. Her early developmental milestones had been normal and she had attended a mainstream primary school. She rode horses and competed in dressage events.

Her problems started at secondary school during which she acquired the label of ‘cerebral palsy’. She fell behind her peers and needed extra educational support in class. She developed problems with concentration and mobility. Her mother noted that she moved her head down when she had to look down, and that she needed to raise objects to eye level to examine them. She became unsteady on her feet but could still walk unaided. At aged 15 years, she moved from secondary school to a facility to provide life skills training for adolescents with special needs. She could read and write at this stage and began to work as a shop assistant.

Over the next few years, she developed posturing of her right foot and fidgeting movements of her mouth, face, arms and legs. She had no difficulties with stiffness, sensory problems, bladder or bowel issues. Her balance worsened and she began to fall. Her speech was stuttering but she remained independent. She became increasingly unsteady and depended more on her mother for support. She acquired a wheelchair. Her speech regressed from being able to talk in sentences to only being able to say a few words.

At the age of 22 years, she presented to her local neurology department with nocturnal generalised tonic-clonic seizures. She had clusters of seizures for every few months and was initially treated with sodium valproate; this was subsequently withdrawn following deteriorating cognition and balance, and levetiracetam started. She became seizure-free after 2 years on 1000 mg daily. Episodes of head drop also started at the same time as the seizures. These occurred after laughter and recovered in seconds. The episodes of head drop decreased at the same time as the seizures were managed. She had no excessive daytime sleepiness, night terrors, dream recall or sleep paralysis. At age 28, she developed swallowing difficulties and started a soft diet. Her mood was stable and there were no obvious symptoms to suggest depression or psychosis.

**Figure 1:**
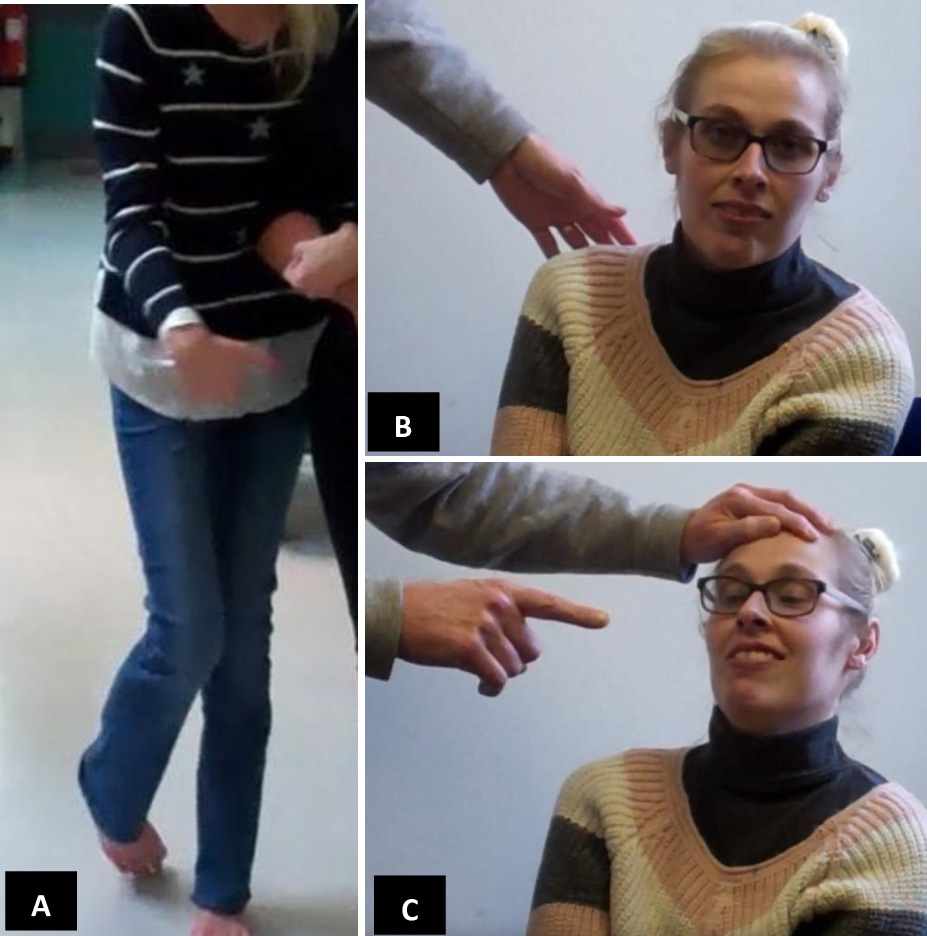
(A) Dystonic posturing of the patient’s right arm and leg. (B) Patient attempting downward gaze followed by (C) this being overcome during fixation while her head is tilted back, demonstrating her supranuclear gaze palsy.

On examination when aged 29, she was alert and responsive. She had an asymmetrical grimace of her face and involuntary movements of her face, arms and legs (figure 1, for examination see online supplementary video). Her speech was slow. She took a few steps with the help of two people but was very unsteady and had dystonic posturing of her arms and legs. She had a vertical supranuclear gaze palsy and a full range of voluntary horizontal eye movement. Horizontal saccades to target and command were slow. There was no spasticity and her reflexes were normal with downgoing plantar responses. Sensation was normal. Her Addenbrookes Cognitive Examination gave a total score of 24/100 with impairments in all domains: attention and orientation 6/18; memory 2/26, fluency 2/14; language 10/26; visuospatial 4/16. She had a normal systemic medical examination and had no organomegaly. Slit lamp examination in the eye department was normal.

10.1136/practneurol-2019-002236.supp1 Supplementary video

She had a mild iron deficiency anaemia. Her normal or negative blood investigations included renal, liver and thyroid profile, C-reactive protein, erythrocyte sedimentation rate, anti-nuclear and related antibodies, immunoglobulins, serum protein electrophoresis, HIV, syphilis and a blood film for acanthocytes. Serum caeruloplasmin, amino acids and very-long-chain fatty acids were normal. Urinary acylcarnitine profile and organic acids were normal.

**Figure 2:**
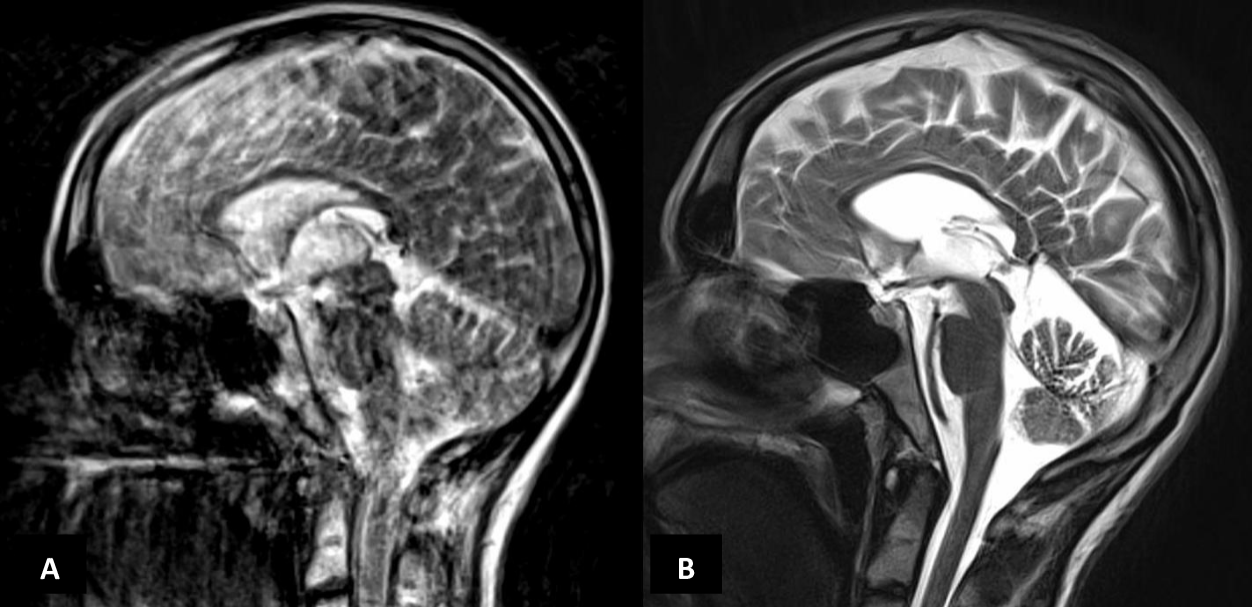
(A) MR scan of brain (T2-weighted sagittal) in 2012 (image quality degraded by movement artefact) and (B) a corresponding section from 2018 showing prominent cerebellar and corpus callosal atrophy.

MR scan of brain showed progressive cerebellar and corpus callosum atrophy over 7 years (figure 2). Cerebrospinal fluid (CSF) studies showed normal cells, protein and glucose (plasma 5.2 mmol/L, CSF 3.6 mmol/L) and negative DNA PCR for *Tropheryma whipplei*. ECG was normal. EEG at 29 years showed slow transients with spike components in the temporal regions but no seizure activity.

## Differential diagnoses

This patient presented with a progressive neurological syndrome that developed over ten years from adolescence, comprising seizures, cognitive deterioration, cataplexy, supranuclear vertical gaze palsy, swallowing difficulties, ataxia and dystonia. This implicates the cerebrum, basal ganglia, cerebellum and brainstem in the disease pathophysiology. Progressive genetic and metabolic conditions were considered most likely.

The individual components of the disorder have a broad differential diagnosis but this combination strongly suggested Niemann-Pick type C. The differential diagnosis of a progressive ataxia with an extrapyramidal syndrome also includes spinocerebellar ataxia type-2, 3, 17, ataxia telangiectasia and neuroacanthocytosis.^1^ We considered Huntington’s disease and dentato-rubro-pallido-luysian atrophy due to the cognitive, cerebellar and extrapyramidal features, but the supranuclear gaze palsy is atypical. Her dystonia and seizures could point to glucose transporter type 1 deficiency; typically, this would be absence seizures starting before the age of 4 years and the normal CSF glucose would go against this. Wilson’s disease can have a variable presentation with ataxia, dystonia and gaze palsy and is of course treatable. There were no Kayser-Fleischer rings but serum caeruloplasmin was also tested. Whipple’s disease can cause an ophthalmoplegia and movement disorder, but there were no gastrointestinal features and CSF PCR was negative. Lysosomal storage disease such as Gaucher’s disease type A can cause all the symptoms our patient described but these patients usually present with an early horizontal supranuclear palsy. The serum oxysterol test for Niemann-Pick type C fell into the 95% limits for the disorder at 61.5 ng/mL (normal range 9.6 to 37, 95% CI for disorder 39.3 to 811.9).

## Discussion

### Niemann-Pick type C

Niemann-Pick type C is a pan-ethnic neurodegenerative disorder caused by the accumulation of glycosphingolipids. Mutations in either the *NPC1* or *NPC2* gene are associated with abnormal endosomal–lysosomal trafficking resulting in the accumulation of lipids in the lysosomes. It has a predicted prevalence as high as 1:19 000–36 000 based on exome sequencing data of known disease-causing mutations.^2^ Its ‘classical’ incidence is approximately 1:100 000.^3^

The clinical spectrum of Niemann-Pick type C ranges from a rapidly progressive neonatal form to a slowly progressive adult-onset neurodegenerative syndrome, and patients can live into the seventh decade of life.^4^ The early onset form usually also presents with cholestatic jaundice, hepatosplenomegaly and/or acute liver failure; these features may all be absent from the late-onset form.

Gelastic cataplexy is more common in early onset forms. However, a consistent feature is a vertical supranuclear gaze palsy, with downward gaze affected initially before vertical gaze. MR imaging of the brain may not help but characteristic findings include cerebellar and corpus callosal atrophy.

### The role of oxysterols in diagnosis

Historically, the diagnosis of Niemann-Pick type C was cumbersome and made using cholesterol esterification studies and filipin staining of cultured skin fibroblasts.^5^ Recently, genetic testing of the *NPC1* and *NPC2* genes is the most widely performed and accessible test. However, in 10% of patients, only one pathogenic mutation can be identified, and new mutations of uncertain significance may be identified in some patients.

Mutations of either gene also affect cellular trafficking of cholesterol, and detecting oxidative cholesterol metabolites can be diagnostic for Niemann-Pick type C. Serum oxysterol can be used as a first-line test with subsequent genetic confirmation and has a positive predictive value of >97%.^6^ It may be elevated in other metabolic disorders such as acid sphingomyelinase deficiency and lysosomal acid lipase deficiency, and to a lesser degree cerebrotendinous xanthomatosis. However, an elevated oxysterol along with classical clinical findings support a diagnosis of Niemann-Pick type C.

### Miglustat as a treatment for Niemann-Pick type C

Although there is no cure for this condition, cohort studies and randomised controlled trials have identified miglustat, a substrate reduction therapy, as a treatment option.^7 8^ In some patients, the drug halts or attenuates disease progression, and it is the first drug that shows both animal and clinical data supporting a disease-modifying benefit for Niemann-Pick type C. Consensus guidelines indicate that the drug should be offered to all patients unless they have a profound dementia resulting in the need for 24 hours care, inability to walk without a wheelchair, complete lack of verbal communication or swallowing difficulties profound enough to require feeding through a percutaneous endoscopic gastrostomy.^9^ Miglustat was initially authorised for use in exceptional circumstances by the European Medicines Agency in 2002 and then following trial data with a broader use from 2009.

## Conclusion

Niemann-Pick type C is a rare lysosomal storage disorder of which all neurologists should be aware, as it is treatable. A progressive vertical supranuclear gaze palsy, gelastic cataplexy, ataxia, dystonia and dementia strongly suggest the diagnosis. Serum biomarkers such as oxysterol have become available since previous reports based on the Filipin test,^10^ which is labour intensive, making diagnosis easier. Currently, there are drug treatment options available and more are being tested in clinical trials that may benefit more patients in the future. An association, Niemann-Pick UK (www.npuk.org), exists to support patients and families experiencing the condition in the UK.

**Key points**

- Cerebral palsy produces a fixed deficit—progressive ‘cerebral palsy’ needs re-evaluation.
- Niemann-Pick type C is a rare cause of a vertical supranuclear gaze palsy and of progressive movement disorder.
- Many patients with this condition have cataplexy.
- Testing for serum oxysterol can give a rapid diagnosis. Miglustat can stabilise the disease and sometimes improve cognitive function and swallowing.
